# Developing an implementation strategy to scale a community-based football intervention for Men's health: an implementation science study

**DOI:** 10.3389/frhs.2026.1835183

**Published:** 2026-07-10

**Authors:** Christopher McDermott, Aisling McGrath, Laura Finnegan, Tom Egan, Eoin King, Rory Sheppard, Noel Richardson, Michael Harrison, Peter Krustrup, Steve Daly, Paula Carroll

**Affiliations:** 1 Centre for Health Behaviour Research, South East Technological University, Waterford, Ireland; 2 Football Research Group, South East Technological University, Waterford, Ireland; 3 Department of Accountancy and Economics, South East Technological University, Waterford, Ireland; 4 National Centre for Men’s Health, South East Technological University, Carlow, Ireland; 5 Department of Sports Science and Clinical Biomechanics, Sport and Health Sciences Cluster (SHSC), Faculty of Health Sciences, University of Denmark, Odense, Denmark; 6 Danish Institute for Advanced Study (DIAS), University of Southern Denmark, Odense, Denmark; 7 Sport and Health Sciences, University of Exeter, Exeter, United Kingdom

**Keywords:** community-based intervention, gender-responsive, health promotion, implementation science, Men's health, recreational football, scalability

## Abstract

**Background:**

Men experience poorer health outcomes than women and are less likely to engage with traditional healthcare services, highlighting the need for gender-responsive, community-based approaches. The Football Cooperative (FC) initiative in Ireland uses recreational football as a socially engaging setting to support men's health and wellbeing. Although football-based initiatives demonstrate strong acceptability and health benefits, translating locally successful models into sustainable systems capable of large-scale delivery remains challenging.

**Methods:**

This study aimed to identify multilevel determinants influencing implementation of the FC initiative and to develop a prioritised implementation strategy to support national scale-up. A two-part qualitative design was employed, comprising (1) multilevel data collection through semi-structured interviews, focus groups, and reflective logs with stakeholders across participant, provider, organisational, and community/system levels, and (2) strategy development and prioritisation through an adapted Delphi consensus process. Data were analysed using a framework-informed approach guided by the Consolidated Framework for Implementation Research (CFIR), and scalability was further examined using the Intervention Scalability Assessment Tool (ISAT).

**Results:**

Findings indicate that implementation of the FC initiative is underpinned by relational facilitators supporting sustained engagement, including psychological safety, inclusive gameplay, and accessibility and operational practicality at participant level. However, while these support local delivery, they do not readily translate to scale. Critical determinants constraining scale-up were identified across ecological levels, including reliance on volunteer coordination and limited role clarity at provider level, informal administrative and digital systems and limited workforce capacity at organisational level, and the absence of formalised governance, cross-sector collaboration, and sustainable funding at system level.

**Conclusions:**

In response, seven implementation strategies were prioritised: maintaining psychological safety and accessibility (participant level); strengthening coordination systems and volunteer capacity and role support (provider level); developing monitoring and digital infrastructure and supporting governance transition and distributed leadership (organisational level); and establishing cross-sector collaboration and funding mechanisms to support scale (community/system level). This study provides a theory-informed, stakeholder-endorsed implementation strategy to support the transition of a community-based men's health initiative from local delivery to scalable systems. The findings contribute to implementation science by demonstrating how multilevel determinant analysis can be translated into prioritised strategies supporting scale-up of gender-responsive interventions.

## 1 Introduction

### 1.1 Men's health in focus

Despite recent medical and social progress, a significant health disparity remains: men in high-income countries, and globally, still face consistently poorer outcomes compared to women (1, 2). This gender-based divide is starkly evident in life expectancy (LE) figures across Europe and Ireland, where men trail women by 5.3 and 3.3 years, respectively (3, 4). This trend, marked by higher premature mortality from non-communicable diseases, injuries (including accidental and traumatic injury), and suicide highlights a persistent inequality (5–8). However, the most socioeconomically deprived groups of men experience some of the poorest health outcomes, including LE differences of approximately 5–10 years and substantially higher premature mortality compared with more advantaged men in Europe (8, 9) and Ireland (10). Successive Irish national men's health policies and action plans (11–13) and the World Health Organisation European Strategy on the Health and Well-being of Men (14) have advocated for a shift toward coordinated, gender-responsive, and multisectoral interventions.

### 1.2 Gender-responsive approaches to Men's health and the role of football in scale-up

An increasing body of evidence demonstrates that gender-responsive men's health interventions are characterised by engaging men through settings and activities that align with masculine identities, social norms, and peer networks, rather than through formal or clinical services (15–19). Men are more likely to participate in health-related activities when these feel familiar, non-stigmatising, and are embedded within everyday routines. This is even more common where participation supports social connection and belonging (17, 20). In this sense, community-based sport/physical activity (PA) interventions and/or sports settings can function as a “hook” for men's health, engaging men through valued social practices before health goals are made explicit (21, 22). We also know that such health interventions for men can significantly improve men's health outcomes (23–25).

The use of football settings (soccer; hereafter referred to as football throughout) has shown great promise; football is a culturally embedded activity with strong social meaning and legitimacy for many men (26, 27). Football settings can reinforce valued masculine identities, facilitate peer support, and provide a social space for engaging with health messages, particularly among men less likely to attend traditional health programmes or settings (18, 28–30). However, it cannot be assumed that any intervention positioned within a football setting will be effective by virtue of the setting itself. For example, the commercial relationships embedded in professional football, including gambling sponsorship, can constrain intervention delivery and complicate assumptions that football club-based approaches are transferable across health issues. Recent evidence from the Football Fans and Betting (FFAB) programme, modelled on FFIT to address gambling harm in professional football club settings, illustrates this: despite strong acceptability among participants, recruitment and retention were substantially undermined by the saturation of professional football's commercial landscape by the gambling industry, with more localised, community-based delivery contexts proving more viable (31, 32).

While there is some ambiguity about the use of football settings for health interventions, there is now a significant body of evidence in support of football itself as a health intervention and that demonstrates that football is, in fact, “medicine” (33, 34). Beyond its social appeal, small-sided recreational football also delivers physical, social and mental health benefits at a relatively low cost, including improvements in psychological wellbeing (35), reductions in anxiety and depressive symptoms (36), and enhanced social connectedness and belonging (37) among previously inactive and socially isolated men. Community-based football programmes have also shown psychosocial benefits for participants experiencing mental health difficulties, including improved confidence, social interaction, and sense of purpose (38). Improvements in aerobic fitness, blood pressure, body composition, and metabolic health are also evident among previously inactive men, men with hypertension and type 2 diabetes mellitus, and overweight and obese adults (21, 29, 33, 39, 40). Professional football clubs have also been used to good effect as delivery settings for health promotion and health awareness campaigns, enabling programmes to engage men through trusted sporting institutions and reach individuals unlikely to access traditional health services (21, 41). For example, “walking football”, a modified, lower-intensity format primarily targeting older adults, has demonstrated physical, psychological, and social health benefits across a growing international evidence base (42). MAN v FAT Soccer, a structured weight-loss programme delivered through football leagues for overweight and obese men, has shown preliminary effectiveness in reducing weight and improving psychological outcomes in feasibility testing (43).

The Football Fans in Training (FFIT) programme is an exemplar evidence-based practice (EBP) for men that uses both “football as an intervention” and “the football setting”. Critically, FFIT, capitalises on fan loyalty and club identity, using them as primary recruitment and engagement mechanisms (44, 45). The 12-week weight loss programme integrates both classroom-based behaviour change programming with pitch-side physical activity across 24 structured sessions, delivered by trained coaches who also were strongly associated with the club. Other gender responsive aspects of the programme included its social dynamics, characterised by banter and peer support (team-mateship), that worked explicitly with masculine norms while FFIT has demonstrated clinically meaningful improvements in weight and health-related behaviours and has scaled successfully across professional club settings (44, 45, 57).

Gender responsive, EBPs such as FFIT (46, 47), Men on the Move [MOM] (48) and Sheds for Life [SFL] (49, 50) have also moved beyond pilot or local single-site delivery and have effectively scaled for the benefit of population health (44, 48, 49). However, this is not commonly achieved. Contemporary implementation science (IS) highlights a persistent research-to-practice gap, often cited as approximately 17 years, although this estimate has been increasingly critiqued as an oversimplification of complex implementation processes (51). Despite this, many EBPs are not successfully embedded or sustained in real-world settings (52–55). Some of the key characteristics of successful scale up of FFIT include positioning its governance within the Scottish Professional Football League (SPFL) Trust, securing funding from the Scottish Government, building capacity among FFIT coaches (56) and continued monitoring and evaluation (57). This approach has enabled FFIT to “scale out” to other population groups (women and men with prostate cancer) to great effect, ultimately creating a positive, long-term impact on the lives of over 13,000 people in Scotland (56). However, initiatives such as SFL, that demonstrate strong acceptability and effectiveness, identified critical threats to long-term sustainability at scale-up, such as institutional capacity and funding constraints, that warrant consistent oversight (49, 50). It is evident, therefore, that while EBPs for improving men's health are emerging, there is a gap in the translation of local EBPs to multi-site scale-up to impact men's health at a population level. This translation requires a rigorously developed, theory-informed, stakeholder-endorsed implementation strategy that is sensitive to factors such as, what constitutes intervention success, delivery infrastructures, and resource constraints (50, 53, 55). One such initiative involving the use of recreational football will now be considered.

### 1.3 The football cooperative (FC) initiative

Football Cooperative (FC) (see https://www.footballcooperative.ie) is an Irish social enterprise (SE) established in 2017, which uses recreational “pick-up” football at two sites as a socially engaging and gender-aligned mechanism to engage men in health and wellbeing practices. The FC initiative was founded not as a clinical or structured health programme, but to create a sustainable, community-based model in which men could participate regularly in football in an inclusive, values-led environment. Participation is open to any adult man and is not predicated on identified health risk; men join through social media promotion and word of mouth, confirm attendance through a simple digital process, and access games for a nominal weekly fee. What distinguishes the FC initiative from commercial recreational provision, such as pay-to-play five-a-side facilities, is its social enterprise ethos, its active governance of shared values including fairness, inclusivity, and community, and its peer-led facilitation model in which volunteer coordinators are themselves players, collectively sustaining a culture of belonging rather than simply administering access to games. The FC initiative model aligns with masculine identities and social norms by prioritising autonomy, camaraderie, informality, and action-based participation, rather than structured or clinical health provision. Men engage through “doing” rather than disclosure, and with banter and shared effort creating a socially acceptable space for enacting health behaviours without stigma (49). In this respect, the FC initiative's engagement mechanism differs fundamentally from professional club-based models such as FFIT: where FFIT leverages fan identity and institutional trust in a professional club as its hook, and delivers structured behaviour change programming within that setting, the FC initiative's hook is the game itself, a culturally familiar, peer-regulated, and locally accessible activity through which informal social, masculine camaraderie, and sustained community connection emerge without structured programming or professional facilitation. The FC initiative operates year-round, delivering games 50 weeks per year across community astro-turf sites. These games are typically two to three evenings per week, with sessions lasting 60–90 min. Games are open to men aged 18 years and over across varied fitness levels and are organised through peer leadership rather than professional facilitation. Delivery is coordinated by Volunteer Game Coordinators (VC), supporting a flexible, low-cost model that promotes shared responsibility and sustained engagement. VCs also participate as players during games while actively and informally facilitating team organisation. Weekly delivery follows a structured coordination cycle led by VCs. The weekly coordination cycle begins with a WhatsApp notification, inviting registered players to opt in via Typeform and secure their spot with a nominal fee of €5. Once registration closes, teams are organised based on playing position, experience and perceived ability. This process helps to maintain balanced games. Games are self-regulated rather than formally refereed, with players and VCs collectively maintaining fair play during matches. Within this model, health and wellbeing are neither defined as targets nor measured through structured clinical outcomes during delivery. Rather, they are understood as consequences of sustained, socially embedded participation: men engage primarily for enjoyment, social connection, and sport, and health benefits accrue through that regular engagement over time (59–61). This positions the FC initiative within the broader evidence base for settings-based health promotion, in which the social and physical conditions of a setting, rather than discrete educational or clinical inputs, function as the primary mechanism of health gain (22, 58).

Evidence from a feasibility study demonstrated that the FC initiative attracts men with multiple modifiable cardiovascular (CVD) risk factors (59) and that at 12 months, improvements were reported in aerobic fitness, waist circumference, CVD risk, and psychosocial health including reduced loneliness and improved sleep quality (60). Notably, while the feasibility study confirmed that the FC initiative attracts men carrying multiple modifiable risk factors (59), this was not by recruitment design; it reflects the broader evidence that gender-aligned, socially embedded participation formats can reach men who would be unlikely to engage with formal health services or structured programming (15, 16, 23). Furthermore, health benefits were cost effective with a Social Return on Investment (SROI) of €17.60 for every €1 invested (61). In light of the evidence base, the FC initiative's advisory board (AB), comprising 46 individuals across 32 organisations spanning sport, health, academia, and community sectors (62), established to support the strategic direction of the SE, advised the scale-up of the FC initiative to extend access and achieve population-level impact.

### 1.4 Study aim

This study aimed to identify multilevel determinants influencing implementation of the FC initiative and to develop a prioritised implementation strategy to support phased replication and longer-term scale-up. Guided by implementation science frameworks, the study focused on translating these determinants into practical, prioritised strategies to support adoption, delivery, monitoring, and sustainability across existing and new FC initiative sites. The specific objectives of this paper are to:

- identify key ecological determinants influencing the implementation of the FC initiative across participant, provider, organisational, and community/system levels;
- develop and prioritise evidence-informed implementation strategies to guide the initial phased replication of the initiative; and
- inform longer-term national scale-up planning.

## 2 Materials and methods

This study is registered with the International Standard Randomised Controlled Trial Number (ISRCTN66120372) and received ethical approval from the South East Technological University Faculty of Health Sciences Research Ethics Committee. All participants provided written informed consent prior to participation. The full study protocol is available in the references section (62).

### 2.1 Researcher positionality

The primary researcher (CMcD), a recreational football player and qualified coach, was embedded within the FC initiative as a participating player during data collection, providing direct experiential insight into the social environment, interactional dynamics, and practical realities of delivery. This position supported a deeper contextual understanding of how participation was experienced and how implementation processes operated in practice. Participation was conducted overtly; players at both sites were informed in advance that CMcD would be attending as both a researcher and a participant, and were invited through the FC initiative gatekeeper to take part in the study.

Such embedded engagement has been shown to enhance alignment between research and practice by supporting contextually grounded interpretation and knowledge exchange (63). In this study, the researcher's subjectivity was therefore considered an important source of analytic insight, informing interpretation of participant experiences and implementation processes rather than functioning solely as a source of potential bias.

To ensure rigour, reflexive practices were incorporated throughout the study. The primary researcher maintained reflective logs documenting observations, assumptions, and analytic decision-making during data collection and analysis. These reflections were discussed regularly with the wider research team, who were not embedded within the FC initiative, to support critical examination of interpretations and maintain analytic credibility.

### 2.2 Theoretical approach

A suite of complementary IS frameworks (see Table 1) was used to guide the study design, data collection, analysis, and strategy development for the FC initiative. These frameworks supported a structured progression from; mapping implementation contexts, to identifying determinants, to developing and assessing strategies for scale-up. Bronfenbrenner's Ecological Model structured stakeholder mapping and sampling across participant, provider, organisational, and community/system levels, ensuring that determinants were examined across multiple layers of influence (64). The Consolidated Framework for Implementation Research (CFIR) informed topic guide development and deductive analysis, supporting identification of barriers and facilitators shaping implementation within and beyond existing FC initiative sites (65, 66)]. Identified determinants were translated into actionable implementation strategies using the PRACTical planning for Implementation and Scale-up (PRACTIS) guide, which structured prioritisation of strategies to enhance adoption, delivery, and sustainability (67). Finally, the Intervention Scalability Assessment Tool (ISAT) was applied to assess readiness for scale across domains including strategic alignment, adaptability, system capacity, and resourcing, informing decisions related to phased replication and longer-term national scale-up (68, 69).

**Table 1:** Implementation science frameworks used to guide study design, analysis, and strategy development.

Framework	Function	Application in this study
Ecological Model, Bronfenbrenner (64)	Process model guiding sampling and stakeholder mapping	Defined four ecological levels (individual, provider, organisational, community/system) for data collection and analysis
CFIR, Damschroder et al. (65, 66)	Determinant framework	Informed interview guides and deductive coding of barriers and facilitators across contexts
PRACTIS, Koorts et al. (67)	Process and planning framework	Structured interpretation of CFIR findings into actionable implementation and scale-up strategies
ISAT, Milat et al. (68)	Evaluative and decision-support tool	Assessed readiness for scale, including feasibility, strategic alignment, and sustainability

### 2.3 Study design

A two-part qualitative design was employed. Part A developed a draft implementation strategy using qualitative data collected within and outside the FC initiative across all stakeholder levels. Part B refined and prioritised the draft strategy using an adapted Delphi process, in which a panel of experts on the AB reviewed the strategy and reached structured consensus on priority actions (70). Details of participant recruitment, data collection, and analysis procedures for both study phases are outlined in Sections 2.3–2.5.

### 2.4 Participants and recruitment

#### 2.4.1 Part A: development of draft implementation strategy

Participants for Part A were recruited to capture perspectives across four ecological levels relevant to implementation of the FC initiative as per Table 1. Sampling aimed to capture perspectives across ecological levels and key implementation roles relevant to implementation of the FC initiative (71). Purposive and snowball sampling were facilitated through the FC initiative and AB gatekeepers. Gatekeepers acted at arm's length, facilitating initial contact only, with no influence over participation decisions and no access to raw data. For further details of participant numbers and characteristics, see Table 2. Within the FC initiative, participants included players, VCs, and the co-founder, who was the sole organisational lead participant. Additional stakeholders at organisational and community/system levels included representatives from national sport, volunteering, and governing sport bodies. Stakeholders outside of the FC initiative involved in football-for-health and sport-for-development initiatives in Scotland, Denmark, Germany, Belgium, and Ireland across all ecological levels were also recruited to inform transferable implementation learning relevant to replication planning.

**Table 2:** Part A participants and CFIR-guided methods informing draft implementation strategy.

Ecological level	Stakeholder group	CFIR domains examined	Data collection (method)	Data collection (personnel; *n*; duration)
Within the FC initiative				
Participant	The FC initiative players (*n* = 33)	Characteristics of individuals; Inner setting; Process	Semi-structured interviews	CMcD, PC, SD (*n* = 18; 23–50 min)
			Ad hoc pitch-side interviews	CMcD (*n* = 15; 5–19 min)
			Reflective logs structured using Rolfe's reflective cycle	CMcD (*n* = 14)
Provider	Volunteer Game Coordinators (*n* = 5)	Inner setting; Outer setting; Characteristics of individuals; Process	Focus groups	CMcD, PC (*n* = 2; 23–64 min)
			Semi-structured interview	CMcD (*n* = 1; 60 min)
Organisational	The FC initiative founder (*n* = 1)	All CFIR domains	Semi-structured interviews	CMcD, PC, AMcG, TE, LF (*n* = 6; 50–103 min over 18 months)
Community/system	Strategic leads from FAI^a^ and UEFA^b^ (*n* = 2)	Outer setting; Process	Focus group	CMcD, PC (*n* = 1; 53 min)
Outside the FC initiative				
Participant	Danish “Football for Life” participants (*n* = 14)	Inner setting; Intervention characteristics; Process	Observational site visits documented via reflective logs structured using Rolfe's reflective cycle	CMcD, PC (*n* = 3)
Provider – CMcD, LF	Hamburg FA^c^ coordinators and volunteers (*n* = 6)	Intervention characteristics; Inner setting; Process	Semi-structured interviews	CMcD, LF (*n* = 2; 28–49 min)
			Ad hoc interviews	CMcD (*n* = 4; 7–10 min)
			Reflective log structured using Rolfe's reflective cycle	CMcD (*n* = 1)
Organisational – CMcD, PC	Volunteer Ireland and Basketball Ireland leads (*n* = 2)	Inner setting; Outer setting; Process	Semi-structured interviews	CMcD, PC, TE (*n* = 2; 56–69 min)
Community/system – CMcD, AMcG	Policy and funding stakeholders (Scottish FA; Danish FA; Hamburg FA; Hypercube)	Outer setting; Characteristics of individuals; Process	Semi-structured interviews	CMcD, PC, LF (*n* = 2; 16–41 min)
			Focus groups	CMcD, LF (*n* = 2; 31–61 min)
			Reflective log structured using Rolfe's reflective cycle	CMcD (*n* = 1)

a FAI, Football Association of Ireland.

b UEFA, Union of European Football Associations.

c FA, Football Association.

All participants met the following inclusion criteria: (1) aged 18 years or older; (2) provided written informed consent; (3) proficient in English; and (4) held a stakeholder role relevant to implementation at one of the four ecological levels within the FC initiative or a comparable football or sport-for-health initiative in Ireland or Europe.

#### 2.4.2 Part B: adapted Delphi study

Part B engaged members of the FC initiative AB who were not part of the research team (*n* = 36). Eligible members were invited to participate by the FC initiative's gatekeeper. Thirty-three of those members (92%) participated in the Round 1 consultation, which is a level of engagement considered appropriate for modified Delphi processes in applied health and policy research (72). Following integration of Round 1 feedback, the revised strategy was circulated to all eligible AB members (*n* = 36) for Round 2 asynchronous review. The participants were then invited to provide comments or edits to the prioritised actions within the strategy. In line with pragmatic consensus approaches used in applied health and policy research, non-response was treated as acceptance of the revised strategy. Round 2 served as a confirmation stage to ensure prioritised actions reflected AB discussion.

### 2.5 Data collection

#### 2.5.1 Part A: implementation strategy development

Data collection for Part A occurred between February 2021 and June 2024. A multi-method qualitative approach was used to capture implementation determinants across ecological levels as per Table 2. With the exception of researcher reflections, all primary data collection was informed by CFIR to support systematic exploration of implementation barriers and facilitators across settings.

Within the FC initiative, at the participant level, semi-structured interviews (*n* = 18) and *ad hoc* pitch-side interviews (*n* = 15) were conducted with FC initiative players to explore experiences of participation and perceived facilitators and barriers to engagement. Interviews were conducted as part of the feasibility study that preceded this study (73) and to avoid over-researching participants, data was reanalysed for this study. *ad hoc* interviews were conducted immediately before or after sessions to capture *in-situ* reflections and minimise retrospective bias. The combination of semi-structured interviews, *ad hoc* pitch-side interviews, and focus groups was designed to generate complementary forms of insight across different contexts and relationships. Semi-structured interviews enabled in-depth, individual exploration of participant experiences, implementation processes, and stakeholder perspectives in a reflective setting. *ad hoc* pitch-side interviews captured immediate, *in-situ* responses before or after sessions, reducing retrospective recall and accessing perspectives that might not surface in a formal interview context. Focus groups were used where collective discussion and shared sense-making among providers or system stakeholders was most likely to surface implementation dynamics, including tensions, shared norms, and negotiated understandings. These data sources were integrated analytically through the CFIR-informed framework approach described in Section 2.6, with data from each method contributing to the same deductive and inductive analytic phases. Furthermore, the primary researcher (CMcD) actively participated in Monday night 90 m FC games at both sites, engaging over an 8-week period at Site 1 between March and May 2023, and over a 6-week period at Site 2 between March and May 2024. In total, 14 reflective logs (CMcD) were generated and captured post games. Logs were structured using Rolfe's reflective cycle and aligned with CFIR domains, documenting observations, reflexive considerations, and analytic decision making to support transparency during data generation and interpretation (74). Logs were discussed with team members on a weekly basis and revised for analysis.

At the provider level, the VCs participated in focus groups (*n* = 2) and an interview (*n* = 1) examining operational practices, delivery challenges, and coordination processes across FC initiative sites.

Within the organisational level, longitudinal insight was generated through a series of semi-structured interviews with the FC initiative co-founder (*n* = 6) and conducted over an 18-month period, enabling examination of organisational development, governance, and scaling considerations as the initiative evolved. A focus group (*n* = 1) at community/system level was conducted with representatives from national and international governing and policy organisations to examine system alignment, funding considerations, and structural barriers to replication.

Outside of the FC initiative, interviews took place with programme coordinators and delivery volunteers (*n* = 6), organisational leads (*n* = 2), and policy stakeholders (*n* = 4). This was alongside observational site visits to Danish programmes (*n* = 2), documented through structured reflective logs (*n* = 3).

In addition to primary qualitative data, a desk-based review of outputs from the preceding FC initiative feasibility study was undertaken to provide contextual grounding for strategy development. All qualitative data was digitally recorded and transcribed verbatim.

#### 2.5.2 Part B: adapted Delphi study

Following synthesis of findings from Part A in April 2025, an adapted Delphi process was conducted in May (Round 1) and June (Round 2) 2025. Round 1 involved facilitated small-group workshops, delivered in person and online, where participants reviewed the draft implementation strategy and prioritised actions using an Eisenhower Priority Matrix based on perceived urgency and importance (75). Specifically, actions rated as both urgent and important were classified as “Prerequisite (Do It Now)”. Actions rated as important but less urgent were categorised as “Prerequisite (Schedule It)”. Strategies considered necessary for stability and optimisation beyond initial replication were designated as “Sustaining Conditions”. Following integration of Round 1 feedback, the revised strategy was circulated to all eligible AB members (*n* = 36) for Round 2 asynchronous review. Participants were invited to provide further comments or edits, with non-response treated as acceptance to support pragmatic consensus-building (72).

Data from Parts A and B were integrated to produce the final, prioritised implementation strategy presented in this paper.

### 2.6 Data analysis

#### 2.6.1 Part A: strategy development

All data were first assigned deductively to CFIR constructs across the five domains: intervention characteristics, inner setting, outer setting, characteristics of individuals, and process (65, 66). Initial construct assignment was led by CMcD and reviewed collaboratively with the research team to support consistency and shared interpretation (as per Table 2).

In a second analytic phase, the primary researcher conducted an inductive, interpretive analysis across the CFIR-coded data to examine patterns, relationships, and underlying influences on implementation. This analysis involved coding across the CFIR-organised dataset to develop higher-order implementation determinants as analytic themes, drawing on but not constrained by individual CFIR constructs. These determinants were discussed with the research team and refined through iterative review.

In a final analytic phase, CFIR-informed analytic summaries were developed to organise and compare the agreed implementation determinants across ecological levels and stakeholder groups, consistent with framework analysis approaches in applied health research (76). These summaries supported further refinement through comparison across data sources and contexts, with emphasis placed on implementation relevance and explanatory value rather than traditional claims of thematic saturation. Identified determinants were then translated into draft implementation actions using the PRACTIS guide, which structured the progression from diagnostic mapping to implementation planning (67).

#### 2.6.2 Part B: adapted Delphi study

Data from the adapted Delphi process were analysed using descriptive and qualitative techniques consistent with applied consensus methods (72). For Round 1, feedback generated during facilitated workshops was collated and synthesised to refine strategy wording, merge overlapping actions, and identify areas requiring clarification. Actions were prioritised using an urgent–important matrix to support structured decision-making.

For Round 2, qualitative feedback from the asynchronous review was integrated to confirm prioritisation and finalise strategy content. Edits were incorporated where provided. The final output was a refined and prioritised implementation strategy endorsed through AB consultation.

The final implementation strategy was then appraised via the ISAT to assess readiness for scale across domains including strategic alignment, adaptability, system capacity, and resourcing (68, 69). The ISAT was initially completed independently by four members of the research team (CMcD, PC, AMcG, LF) and drawing on multiple information sources, as per ISAT guidance, domain ratings were informed by outcome and cost-effectiveness evidence (59–61), the multilevel qualitative determinant synthesis, and stakeholder priorities identified through the adapted Delphi process. Following independent scoring, researchers met to review and agree domain classifications through structured discussions ensuring that ratings were grounded in empirical and stakeholder-derived evidence rather than individual opinion. The resulting appraisal was then circulated to other team members (NR, MH, TE) as well as key AB members representing sporting and voluntary organisations (*n* = 3) resulting in a total of 14 contributors to the final assessment. In line with the ISAT development guidance, the tool was applied as a structured decision-support framework to inform scalability planning rather than as a standalone quantitative metric.

## 3 Results

Results are presented in accordance with the PRACTIS guide, which involves four steps: characterising the implementation setting, identifying and engaging key stakeholders, identifying contextual barriers and facilitators, and addressing barriers to implementation. The findings are presented as a synthesis of multi-level implementation determinants which were translated into implementation strategies (PRACTIS Step 4). Scalability was appraised using the ISAT. These inform the identification of the contextual conditions and implementation requirements relevant to potential scale-up of the FC initiative.

### 3.1 Characterising the implementation setting (PRACTIS Step 1)

Consistent with PRACTIS Step 1, the implementation setting of the FC initiative was characterised to clarify the contextual parameters within which delivery occurs and to identify features requiring preservation during future scale-up. This step describes the delivery environment within which subsequent scalability judgements were formed. Rather than functioning as a fixed innovation with tightly standardised components, delivery of the FC initiative was observed to evolve as a values-led community model shaped through ongoing interaction between participants, VCs, and local delivery environments. This helped distinguish core elements of the initiative from those adaptable across settings. The FC initiative's founding values of fairness, respect, inclusivity, and community, its open recruitment model through social media and word of mouth rather than clinical or risk-based referral, and its intended outcomes of sustained social engagement and associated health benefit through participation are described in detail in Section 1.3; Section 3.1 focuses on how those values and structures manifested in the implementation setting characterised here.

Delivery was characterised by a socially embedded participation model centred on weekly recreational football delivered outside formal league structures and supported through informal facilitation practices. VCs played a central role in sustaining participation culture and operational continuity across both sites. The VCs also participated as players during sessions while informally facilitating game organisation and adherence to the initiative's core values.

Weekly delivery follows a structured coordination cycle led by VCs. Prior to each game, a notification is circulated to registered participants inviting them to opt in and confirm attendance through a small participation fee. Once registration closes, teams are organised based on playing position, experience, and perceived ability in order to maintain balanced games. The VCs then oversee delivery of the session and support adherence to FC initiative values of fairness, respect, and inclusivity. Games are self-regulated rather than formally refereed, with participants and VCs collectively maintaining fair play during matches.

The defining characteristics of the FC initiative setting are summarised using the PRACTIS Five Ps framework (Table 3), capturing how interactions between people, place, process, provisions, and guiding principles collectively shape delivery conditions. Stakeholder engagement indicated that these elements operate interdependently rather than in isolation, with participant expectations, volunteer facilitation practices, and organisational supports jointly influencing delivery feasibility and continuity. Collectively those parameters illustrate the FC initiative as a socially embedded delivery model in which cultural norms, facilitation practices, and operational routines support sustained engagement and provide the foundation for the subsequent analysis.

**Table 3:** PRACTIS five Ps applied to the FC initiative setting.

PRACTIS parameter	Definition	Description of the FC initiative setting
People	Who the FC initiative engages and who is involved in delivery.	The FC initiative engages adult men across a wide range of ages, backgrounds and ability levels who seek an enjoyable football experience in a welcoming and inclusive environment. Participants in the feasibility study were typically middle-aged (34.7 ± 8.5 years) with body mass values in the overweight range. VCs play a central role in setting the tone, welcoming participants, and supporting positive interaction, which helps maintain a consistent cultural atmosphere. Participants emphasise belonging and routine, and value being able to take part outside formal league structures while still playing fair, enjoyable and competitive games. This environment is sustained through ongoing interaction between participants and VCs, where facilitation practices and shared expectations shape the conditions for continued engagement.
Place	The physical and social environments where delivery occurs.	Delivery takes place in community-based AstroTurf facilities that offer an informal setting outside formal club structures, which participants experienced as relatively accessible and low in performance-related pressure, though cost and the commercial nature of some venues can present barriers, and some traditional club settings may offer comparable or greater social infrastructure. These venues support evening participation that fits with everyday routines. The setting encourages informal social interaction and reduces barriers often associated with performance-oriented or membership-based environments. However, stakeholders noted that longer-term security of access requires formal site agreements, as continued delivery currently relies on negotiated facility arrangements at each site.
Process	The workflow, routines and delivery structure through which the initiative operates.	The FC initiative operates through a consistent weekly cycle that includes communication with players, opt-in confirmation for games, balanced team selection, and values-led facilitation aligned with fairness, respect, inclusion and community. Communication and coordination are managed using practical digital tools, including WhatsApp messaging for updates, Typeform systems for opt-in and attendance tracking, and site-specific payment arrangements such as Revolut transfers. These routines stabilise delivery and support regular participation; however, reliance on WhatsApp communication and Revolut payments may present limitations for scaling due to GDPR considerations and the absence of integrated financial or participant management systems.
Provisions	The resources and supports required for implementation and sustainability.	Delivery relies on access to affordable facilities, appropriate equipment, insurance cover, and a committed VC group to organise and facilitate games. Operational delivery also requires ongoing time for communication, administration, and on-the-ground facilitation to maintain game quality and consistency. Stakeholders noted that continued reliance on volunteer coordination and affordable facility access may present capacity challenges if the FC initiative expands to additional sites.
Principles	The core ethos and values that guide delivery and participant experience.	The FC initiative is grounded in fairness, respect, inclusion and community. These principles shape expectations for behaviour, guide team balancing and session facilitation, and support mixed-ability participation. The approach prioritises enjoyment and positive social connection, and these principles underpin the core logic of the FC initiative's delivery. Stakeholders also noted that maintaining inclusivity sometimes requires flexibility in participation processes, which can create operational challenges when players join sessions without completing the standard registration cycle.

These contextual characteristics informed subsequent scalability appraisal, particularly shaping ISAT assessments relating to delivery feasibility and workforce capacity.

### 3.2 Stakeholder identification and engagement (PRACTIS Step 2)

Our analysis of stakeholder engagement centred on identifying the key figures driving implementation and understanding how their collaborative input shapes the roadmap for scale-up. Stakeholders contributed to implementation decision-making through a co-design process embedded within the FC initiative. By gathering perspectives across ecological levels, we gained a deeper understanding of everything from the daily experience of players to the broader complexities of system alignment and governance. Players within the participant level described shared expectations within weekly games, emphasising responsibility and mutual accountability between participants:

“You notice who can take responsibility and who you can rely on when it's not going your way”. (Participant)

This highlights the role of peer-regulated norms in sustaining delivery quality. During games these expectations are informally reinforced by VCs, who participate in matches while facilitating fair play and adherence to the initiative's core values.

VCs described the operational work underpinning weekly sessions, noting that participation often appeared simple to players but relied on ongoing coordination behind the scenes:

“Someone has to keep the booking going, get the balls, the bibs, make sure people are turning up. It seems effortless to us [as players], but I know someone at organisational level is putting work in to make it happen”. (Volunteer Game Coordinator)

This illustrates the hidden coordination burden sustaining delivery. While participation appears straightforward, implementation relies on ongoing coordination work by VCs. At organisational level, stakeholders described the development of formal governance and operational structures designed to stabilise delivery and support expansion across sites:

“We established an advisory board… introduced a rulebook… safeguarding and disciplinary procedures… and began formalising coordinator roles and responsibilities”. (Organisational stakeholder)

These developments indicate an ongoing transition from informal, founder-led coordination toward structured organisational governance. Institutionalising roles, procedures, and accountability mechanisms was viewed as necessary to enable replication beyond locally sustained delivery.

Community/system level stakeholders identified the absence of formal representation for recreational football within existing governing structures:

“The recreational game was not represented… having a voice somewhere [in NGB] of recreational football would be the solution”. (Community/System stakeholder)

This reflects limited institutional recognition of recreational participation formats within national football systems. Stakeholders viewed formal recognition within national governing structures as important for legitimacy and policy alignment.

More broadly, football was framed as a vehicle for health promotion and social connection aligned with wider participation and wellbeing priorities:

“I think we need to see football as an object that gets people active and brings people together… football is the reason, or the hook, to do what we need to do”. (Community/System stakeholder)

This framing positions the FC initiative within broader public health and participation agendas, reinforcing strategic alignment with external stakeholders, factors that informed ISAT assessments relating to intervention relevance and system fit.

Across the ecological levels, stakeholder engagement demonstrated that implementation conditions operate interdependently. Participant culture, facilities access, IT infrastructure, volunteer capacity, organisational governance, and system recognition interact to shape delivery feasibility. These insights informed the synthesis of multi-level determinants presented in PRACTIS Step 3.

### 3.3 Synthesis of multi-level implementation determinants (PRACTIS Step 3)

#### 3.3.1 Determinants across ecological levels

Analysis examined determinants influencing implementation of the FC initiative across participant, provider, organisational, and community/system levels. Across stakeholder accounts, determinants were rarely experienced as isolated barriers or facilitators within single ecological domains. Instead stakeholders described interrelated influences in which delivery practices, organisational arrangements, and broader system contexts collectively shaped implementation outcomes. For example, organisational coordination structures influenced provider workload and role sustainability; provider facilitation practices shaped participant experience and retention; and system-level relationships affected organisational capacity, legitimacy, and longer-term sustainability. These accounts indicated that implementation outcomes emerged through interaction between ecological levels rather than from discrete determinants operating independently.

Table 4 presents determinants identified across ecological levels, retaining the detail of stakeholder accounts relevant to implementation planning and strategy development. While determinants are presented discretely for analytical clarity, stakeholder narratives demonstrated that their influence was relational and cumulative. Determinants operating at one level frequently generated consequences at others, reinforcing or constraining implementation across the system.

**Table 4:** Determinants influencing implementation across ecological levels.

Ecological level	Determinant	Description
Participant	Social connection, psychological safety and belonging	The extent to which participants experience the FC initiative as a welcoming and supportive environment, including feeling comfortable, accepted, and able to participate without fear of judgement.
	Accessibility, practical fit and life pressures	Practical factors shaping attendance and retention, including timing, location, cost, competing commitments, and how easily games fit into weekly routines.
	Ability fit, challenge and health management	How well the physical demands and mixed-ability context align with participant capability and confidence, including perceptions of safety, challenge, fitness level, and ability to manage injury or health concerns.
	Motivation, confidence and habit formation	Psychological and behavioural factors influencing continued engagement, including confidence to attend, motivation to participate, and development of regular attendance habits over time.
	Enjoyment and playfulness	The degree to which games are experienced as enjoyable and engaging, supporting ongoing participation through fun, informal play, and positive game experiences.
Provider	Communication, coordination and logical systems	The systems and processes that support weekly delivery, including communication with participants, coordination of attendance, and consistent administration (for example sign-up, payments, and team organisation). While participants reported satisfaction with existing communication tools, current systems (e.g., WhatsApp messaging and informal payment transfers) present limitations for organisational coordination, financial security, and GDPR compliance if the initiative expands to multiple sites.
	Cultural cohesion, inclusion and fairness	How providers sustain an inclusive mixed-ability environment, including managing behaviours and expectations to protect fairness, respect, and a positive participation climate.
	On-pitch facilitation and adaptive delivery	Provider capability to manage delivery in real time, including balancing teams, adapting formats to the group, maintaining flow, and responding to varied ability levels and participation needs.
	Volunteer motivation, identity and wellbeing	Factors influencing continued provider involvement, including motivation to support the FC initiative, role identity, workload pressures, and supports needed to sustain volunteer contribution over time.
	Partnerships and organisational support	The extent to which providers have support from organisations and partners (for example facility access, equipment, coordination support), and clear backing that enables reliable weekly delivery.
Organisational	Strategic leadership, culture and vision	Organisational leadership and clarity of purpose for the FC initiative, including alignment on what it is, what it aims to achieve, and how growth and delivery should be guided.
	Organisational systems and communication	Internal systems that support game delivery and scale-up, including communication structures, coordination processes, and mechanisms that enable consistency across sites.
	Organisational capacity (internal delivery capability)	The internal staffing, time allocation, and operational capacity within the FC initiative required to coordinate delivery and reduce over-reliance on individual volunteers and the organisational lead as the initiative expands.
	Learning transfer and knowledge transfer	The ability to capture and share learning across sites, including resources, guidance, and support processes that enable consistent delivery as the FC initiative grows.
	Partnerships and external engagement	Organisational relationships that support delivery and scale-up, including engagement with facilities, community partners, and stakeholders who can provide practical support and legitimacy.
	Funding, resource and management	The availability and management of funding and resources required for the FC initiative delivery and scale-up, including planning for staffing, systems, and ongoing operational needs.
Community/System	Accountability and governance structures	Leadership, transparency, and shared responsibility across governing and community layers, including distributed leadership ethical stewardship, and clarity of roles at organisational and system level.
	Cultural and societal context	Broader cultural values and social expectations shaping engagement, including football's symbolic role, community identity, and cultural resonance that sustains participation and support.
	Education and workforce development	Training, professionalisation, and skill-building for those facilitating recreational football, including development of non-traditional roles beyond coaching.
	Equity, diversity, and inclusion	Commitment to fairness, representativeness, and accessibility across programmes and governance, linked to legitimacy and alignment with equality goals.
	Evaluation, research, and evidence integration	Data generation and use of evidence to inform design, justify funding, and demonstrate impact, including partnerships that support evidence-led dialogue and learning.
	Funding and resource environment	Stability, allocation, and accessibility of financial resources across community and national levels, including co-funding and multi-source arrangements.
	Infrastructure and facilities access	Availability of spaces, materials, and logistics needed to deliver the FC initiative, including pitch access, scheduling, and equipment frameworks that enable participation.
	Institutional support and policy infrastructure	The readiness of national governing bodies, public agencies, and sector institutions to recognise, support, and integrate recreational football within formal governance and policy structures.
	Partnerships and networks	Collaboration across sport, health, education, and private sectors, including formal alliances and informal grassroots relationships that support delivery and scale.

Community/system stakeholders also highlighted how partnerships and funding arrangements influenced perceptions of organisational stability and future scale-up potential. For example, the FC initiative is currently supported through funding administered via Rethink Ireland, with contributions from multiple partners including the Football Association of Ireland. While these relationships were viewed as important signals of institutional support, stakeholders also noted that long-term sustainability would depend on strengthening organisational capacity and governance structures as the initiative expands.

This cross-level synthesis informed the scalability profile later reflected in the ISAT appraisal (Section 3.5), where qualitative insights regarding workforce capacity, IT infrastructure, site access, governance arrangements, cultural fidelity, and system alignment were translated into structured scalability judgements. The synthesis below therefore examines interactional patterns between determinants, identifying mechanisms through which influences across ecological levels combined to shape implementation dynamics within the FC initiative.

#### 3.3.2 Psychological safety and relational fidelity

Participant accounts consistently identified psychological safety as central to sustained engagement with the FC initiative. Participation depended on an environment where ability differences were accepted and enjoyment was prioritised over performance or competition. One participant stakeholder described games as “*a safe space to enjoy football without too much pressure*”. Participants contrasted this atmosphere with previous football environments perceived as overly competitive or exclusionary, indicating that reduced performance expectations enabled continued participation among participants who might otherwise disengage from organised sport.

These experiences were actively produced through facilitation practices at provider level rather than occurring incidentally. VCs shaped the game environment through intentional team balancing and informal oversight that maintained fairness and inclusion during games. As one Provider stakeholder explained:

“One thing [Organisational Lead] does really well is balancing the teams… making sure everyone gets involved”.

Providers also described intervening when play became too competitive to preserve the intended tone of games:

“Some people get too excited, and that's when you have to step in, calm things down, and be a voice of reason”. (Provider stakeholder)

In practice, creating psychological safety operated as a cross-level mechanism linking provider facilitation practices with participant retention. Relational qualities of delivery, including inclusive language, behavioural regulation, and balanced play, function as core components of implementation fidelity rather than peripheral aspects of initiative experience.

Stakeholders further highlighted that maintaining this culture represents a central challenge for scale-up. While cultural principles are recognised within the initiative, stakeholders noted that they have not yet been formally mapped or operationalised across sites, indicating a requirement for clearer articulation and transfer mechanisms during expansion of the FC initiative. The Organisational stakeholder noted:

“The culture is a key cog in the successful implementation of Football Cooperative at scale. It needs to be mapped, understood, and continually reinforced across sites”.

Psychological safety therefore functions as both a primary engagement mechanism and a critical fidelity condition for scalability, a gap reflected in subsequent ISAT assessments relating to implementation fidelity and workforce capacity. Expansion depends not only on replicating delivery structures but on sustaining the relational practices through which inclusive participation environments are both created and maintained. However, the findings also point to an important qualification: football spaces are not inherently inclusive or health-promoting, and hegemonic masculine norms within football culture can reinforce competitive, exclusionary, or risk-taking behaviours that work against health. The active facilitation practices of the FC initiative, including values-led conduct, mixed-ability team balancing, and VC-regulated behavioural norms, represent a deliberate departure from such norms rather than an assumption that football participation is beneficial by default. This is consistent with evidence suggesting that masculinities do not operate uniformly across intervention contexts, and that professional football settings in particular can carry commercial and cultural pressures that constrain health-promoting delivery (31, 32). The FC initiative's community-based, non-commercially embedded context, and its active governance of inclusive norms, are therefore implementation conditions rather than incidental features.

#### 3.3.3 Operational infrastructure and capacity building

Implementation of the FC initiative relies on coordination practices that enable flexible local delivery but requires substantial organisational effort. Across provider and organisational stakeholder accounts, weekly delivery appeared operationally simple from a participant perspective while dependent on significant behind-the-scenes coordination undertaken by VCs and the organisational lead solely on one site.

The organisational stakeholder described responsibilities extending beyond game facilitation to include communication with participants, scheduling, administration, and reporting tasks necessary to sustain weekly sessions:

“Right now, Game Coordinators handle everything, from game notifications to post-game reports. That is a huge workload”. (Organisational stakeholder)

This reflects the concentration of operational responsibility within a small number of volunteer roles, highlighting workforce capacity as a central implementation determinant.

While these processes enabled reliable delivery, providers did also describe operational strain associated with coordinating games week to week, highlighting how delivery depended heavily on the judgement and effort of individual organisers:

“The game never ends up as planned on paper. The team sheets sent out early are never accurate. Numbers are either under or over, and you never have the right people. (Organisational lead) has to deal with a lot of people coming and going, so it's tough on him. Maybe he needs extra help or needs to be stricter”. (Provider stakeholder)

The contrast between the apparent simplicity of participation and the complexity of organising games illustrates an important cross-level dynamic. Weekly delivery relied heavily on the continued effort and decision-making of a small number of organisers, exposing a potential vulnerability where operational continuity depended on individual capacity rather than structured systems.

As participation increased, stakeholders described growing operational strain associated with reliance on manual systems and individual effort. Coordination demands accumulated across communication, attendance tracking, payments, and late adjustments, contributing to fatigue among those responsible for delivery:

“…naturally, game delivery and participant coordination fatigues people… There are headaches around it in terms of getting the links out, game notifications, getting people confirmed, collecting game fees, preparing team sheets, and then last-minute changes… So just so you can see that [workload] fatigues people”. (Organisational stakeholder)

This account indicates a vulnerability where delivery continuity depends on sustained individual capacity rather than distributed or system-supported processes. This concentration of labour informed ISAT considerations relating to implementation infrastructure and workforce sustainability.

The organisational stakeholder identified the absence of structured guidance and training as a constraint for replication, noting new coordinators currently rely on informal and *ad hoc* learning rather than standardised preparation:

“This [developing structured training] gives us an opportunity to give them a framework to follow, because just telling a volunteer to go organise kickabouts isn't enough. They need a blueprint. They need a framework to work to”. (Organisational stakeholder)

This suggests that codifying delivery processes and providing structured training may strengthen transferability across sites, reducing reliance on tacit knowledge and individual experience.

The findings position operational infrastructure as a cross-level implementation mechanism linking workforce capacity with sustainability of the FC initiative. Stakeholders described potential scale-up of the FC initiative as dependent on strengthening administrative systems, clarifying VC roles, and enhancing organisational supports in ways that reduce reliance on voluntary labour while maintaining the accessibility and informality valued by participants. Capacity building reflects a transition from delivery sustained through individual effort toward more system-enabled implementation capable of supporting expansion across sites, including the development of integrated IT systems that could streamline communication, payment processing, and automated team selection for Volunteer Game Coordinators.

#### 3.3.4 Governance transition and distributed leadership

The FC initiative was developed within a relational, locally driven governance model that supported early delivery; however, this was viewed as creating challenges for sustainable expansion. Organisational and community/system stakeholders described scalability as requiring stronger governance arrangements capable of distributing responsibility beyond the current model, which is largely sustained by a single organisational lead, reflecting informal leadership structures that are over-reliant on one leader, and supporting integration within established institutional systems (e.g., FAI Club Mark club structures), factors that informed ISAT assessments relating to governance readiness and implementation infrastructure.

Community and system stakeholders emphasised that participation-focused and recreational football has historically received limited institutional support within national and European governance structures. This absence of dedicated funding, staff, and governance mechanisms was seen as restricting recognition of initiatives operating outside traditional competitive pathways. One stakeholder explained:

“When you look at UEFA's perspective and ask, ‘What deliberate actions are being taken to support or recognize recreational football?’ there's nothing. No finance, no staff, no governance”. (Community/System stakeholder)

This absence of institutional structures positioned the FC initiative within an emerging policy space in which development relied heavily on advocacy and relationship-building rather than established organisational pathways within governing bodies e.g., the FAI. Governance engagement was therefore described as enabling not only recognition but also longer-term planning and institutional support. In this context, stakeholders emphasised the importance of moving toward a distributed leadership structure capable of supporting coordination, advocacy, and strategic development as the initiative expands.

Community/system actors further highlighted that scaling such initiatives depends on engagement at senior decision-making levels capable of influencing strategic priorities and resource allocation:

“For this to really have an impact, it needs to be taken seriously in the football context… the conversation absolutely needs to happen at Senior Management level”. (Community/System stakeholder)

These accounts reflected a shift from locally sustained leadership toward governance arrangements distributing responsibility across organisational and system actors. Stakeholders described formal representation and senior-level engagement as mechanisms supporting transition from individually driven coordination toward structures capable of sustaining workforce development, partnership investment, and longer-term implementation planning.

Governance therefore functions as a cross-level implementation mechanism linking system legitimacy with organisational sustainability. Early implementation of the FC initiative was enabled through flexible coordination; however, continued growth was described as dependent on clearer governance positioning, shared leadership responsibilities, and stronger institutional alignment supporting replication beyond local delivery contexts. Governance transition therefore represents a key condition shaping scalability, reflecting movement from individually sustained coordination toward distributed leadership supported through formal recognition and engagement within wider football and health systems. Institutional integration was framed not as a departure from the FC initiative's community-based ethos but as a mechanism for sustaining the model as participation expands.

#### 3.3.5 Institutional integration and funding scale

Implementation of the FC initiative was shaped by local delivery conditions as well as its integration within wider sport, health, and policy systems. Across stakeholder accounts, scalability was described as dependent on positioning the initiative within multi-sector partnerships capable of supporting coordinated planning and long-term investment.

At present, the FC initiative operates primarily as a standalone community initiative delivered through locally organised games. While some site-level collaboration has occurred, including engagement with an IPAS accommodation centre, formal partnerships with health or policy organisations have not yet been systematically established.

Stakeholders nevertheless emphasised that collaboration across agencies could strengthen implementation feasibility by aligning the FC initiative with existing health promotion and community development priorities:

“Organisations like the Health Service Executive, Family Resource Centres, and Public Participation Networks could help signpost men towards our games”. (Organisational stakeholder)

These accounts reflected stakeholder perceptions of how the initiative could be embedded within existing participation and wellbeing infrastructures rather than describing partnerships already operating at scale. Stakeholders described engagement with organisations including the Health Service Executive, Local Sports Partnerships, and community development networks as potential mechanisms through which the FC initiative could contribute to existing health and participation agendas:

“Through building networks and partnerships, we've been able to show that we actually feed into these initiatives rather than compete with them”. (Organisational stakeholder)

Alongside institutional alignment, stakeholders identified evidence generation as a mechanism enabling transition from locally valued practice to system-supported investment. Evaluation findings facilitated evidence-informed dialogue with policymakers and funders:

“Now, with the SROI study, we're finally getting evidence-based confirmation that yes, this model has significant population health benefits and a strong return on investment”. (Organisational stakeholder)

Evidence therefore acted as a bridge between delivery experience and funding discussions, helping justify consideration of expansion beyond locally sustained provision. However, stakeholders also emphasised that scaling the model would require coordinated investment structures rather than reliance on individual leadership or short-term arrangements:

“I think it would be very hard to get a [named individual] at scale. That requires investment”. (Community/System stakeholder)

Across accounts, institutional integration functioned less as a current feature of delivery and more as a proposed mechanism for supporting long-term sustainability. The argument advanced here is not that formalisation of the participation culture is desirable or necessary, but that formalisation of organisational structures, governance arrangements, and coordination systems is required to sustain and replicate the conditions that make informal, inclusive participation possible. In this sense, governance structures, distributed leadership, and digital coordination systems are mechanisms for preserving community ownership and local responsiveness at scale: the scaffolding that enables delivery, rather than a replacement for the relational culture that defines it. Stakeholders consistently linked the future scalability of the FC initiative to stronger alignment with system-level priorities, institutional partnerships, and funding pathways capable of supporting workforce development, infrastructure investment, and coordinated expansion across regions.

Following synthesis of determinants across ecological levels, implementation strategies were developed to address priority influences on scalability identified through PRACTIS Step 3. The resulting strategies are presented below in accordance with PRACTIS Step 4.

### 3.4 Prioritised implementation strategies to address scalability determinants (PRACTIS Step 4)

Building on the cross-level determinants identified in Section 3.3, implementation strategies were prioritised to address key barriers and enabling conditions influencing scalability of the FC initiative (50). As per PRACTIS Step 4, these strategies were developed to strengthen areas of implementation vulnerability identified through the determinant synthesis and to support replication while maintaining delivery fidelity across settings.

Table 5 presents the prioritised implementation strategies mapped to their corresponding determinants and anticipated implementation outcomes (50, 77). The table synthesises actions across participant, provider, organisational, and community/system levels, demonstrating how constraints relating to workforce capacity, governance arrangements, operational infrastructure, cultural fidelity, institutional alignment, and access to suitable facilities were translated into structured implementation responses.

**Table 5:** Prioritised implementation strategies mapped to determinants and implementation outcomes across ecological levels (PRACTIS Step 4).

Mechanism & determinants	Implementation outcome(s) (Proctor, 2011)	Actionable implementation strategies	Priority to scale
Participant Level			
**Accessibility and Operational Practicality** Life pressures, practical fit, motivation, awareness, reach	**Feasibility:** The extent to which a participant can successfully carry out attendance within a busy schedule.	Retain evening scheduling structures that align with identified participant life stage demands and competing commitments.	**Prerequisite (Do It Now)** Critical for converting interest into a weekly habit despite life pressures.
		Formalise simple, low-burden sign-up and communication systems to reduce friction in weekly participation.	
		Maintain low-cost participation structures while monitoring affordability barriers across sites.	
**Psychological Safety and Relational Fidelity** Social connection, belonging, ability fit, enjoyment, safety	**Acceptability:** Perception that the game environment is satisfactory.	Define minimum delivery standards to maintain psychological safety and inclusive participation, including clear expectations regarding contact rules and respectful communication.	**Prerequisite (Do It Now)** Essential to prevent initial intimidation and support early entry.
	**Appropriateness:** Perceived fit for the player's specific fitness/skill level.	Formalise team-balancing procedures to support mixed-ability participation and perceived fairness across sessions.	
**Social Support and Institutional Inclusion** Inclusion, peer support, commitment, diversified reach	**Sustainability:** Maintenance of participation over time; transition from a pilot habit to a stable routine.	Develop structured referral and outreach pathways with community and inclusion-focused organisations to enhance diversified reach.	**Sustaining Condition (Schedule It)** Necessary for long-term retention and reducing participant drop-off.
		Introduce light-touch follow-up mechanisms to support re-engagement following missed sessions.	
		Facilitate periodic social interactions beyond gameplay to reinforce peer connection and routine participation.	
Provider Level			
**Operational Infrastructure and Digital Systems** Coordination systems, administrative burden, session reliability	**Feasibility:** The extent to which delivery can be successfully carried out without fatigue.	Implement simple digital coordination tools to support weekly communication, sign-up, scheduling, fee collection and automated team selection across sites.	**Prerequisite (Do It Now)** Critical to address volunteer burnout before scaling to new sites.
	**Implementation Cost:** Reducing the time-cost of volunteer labour.	Define repeatable weekly delivery workflows to support consistent session implementation across sites.	
**Capacity Building and Role Support** Volunteer training, role identity, workload distribution, confidence	**Adoption:** The initial decision and continued intention to try or lead sessions.	Develop structured training and induction processes to support VC role clarity, confidence, and delivery competence.	**Prerequisite (Do It Now)** Essential for learning transfer and maintaining quality across sites.
	**Fidelity:** Ensuring the game is delivered as intended by developers.	Introduce shared facilitation arrangements to distribute workload and reduce reliance on single coordinators within sites.	
**Cultural Cohesion and Value Facilitation** Inclusion, fairness, on-pitch facilitation, mixed-ability delivery	**Fidelity:** The degree to which the non-judgmental environment is protected.	Define minimum facilitation expectations supporting inclusive mixed-ability delivery and early behavioural regulation during play.	**Sustaining Condition (Schedule It)** Necessary to protect the relational fidelity as the network grows.
	**Acceptability (Provider):** The VCs satisfaction with the delivery model.	Establish peer learning and reflective practice mechanisms to support ongoing adaptation and shared problem-solving among coordinators.	
Organisational Level			
**Governance Transition and Distributed Leadership** Strategic leadership, oversight, role clarity, accountability	**Sustainability:** The extent to which the SE becomes institutionalised in stable operations.	Introduce distributed leadership arrangements that distinguish strategic governance roles (executive oversight) from operational leadership roles responsible for site coordination, volunteer support, and delivery management, including the introduction of paid executive and operational roles to support scaling.	**Prerequisite (Do It Now)** Critical to reduce over-reliance on the Co-Founder and build organisational resilience.
	**Feasibility:** Organisational capacity to manage expansion without failure.	Clarify governance roles and oversight mechanisms to support accountability, risk management, and strategic decision-making.	
		Develop structured site replication guidance distinguishing core components from adaptable elements to support fidelity during scale-out.	
**Infrastructure for Scale and Capacity Building** Funding diversity, IT capacity, learning transfer, site supports	**Implementation Cost:** Managing the start-up and incremental costs of replication.	Develop a funding and infrastructure strategy aligned with expanding the workforce, securing long-term facility access agreements with pitch providers to stabilise weekly delivery across sites, developing appropriate IT infrastructure to reduce workload burden, and ensuring fidelity of replication through structured capacity building.	**Prerequisite (Do It Now)**
	**Sustainability:** Maintenance of the SE via diversified, long-term funding.	Introduce an integrated digital coordination system to support communication, registration, payments, and team allocation across sites.	
**Infrastructure for Monitoring Impact and Implementation at Scale**		Develop scalable monitoring and data systems capable of supporting multi-site coordination, evaluation, and organisational learning.	**Sustaining Condition (Schedule It)** Necessary to move from a small EBP to a stable national entity.
		Embed routine monitoring and impact documentation processes to support strategic decision-making and external accountability.	
Community/System Level			
**Cross-Sector Collaboration and Funding Scale** Local delivery pathways, co-funding, resource mobilisation	**Penetration:** Number of eligible providers (LSPs/Clubs) delivering the service.	Formalise cross-sector referral pathways linking community, health, and sport organisations to support participant recruitment and service integration.	**Prerequisite (Do It Now)** Essential to secure the infrastructure for scale and legitimise the model within existing ecosystems.
	**Feasibility:** Practicality of multi-sector coordination.	Develop multi-source funding arrangements aligned with cross-sector delivery responsibilities to reduce reliance on short-term funding cycles.	
**System-Wide Capacity and Scaling Supports** Workforce development, scaling supports, shared ownership	**Adoption:** Intention of new community partners to try or adopt the FC initiative.	Define minimum system-level requirements supporting consistent site initiation across delivery contexts.	**Prerequisite (Schedule It)** Critical for learning transfer so delivery quality is maintained as the network expands beyond the founding sites.
	**Fidelity:** Ensuring consistency as the model is delivered in new contexts.	Recognise and resource recreational facilitation roles within governing body participation structures to support sustainable workforce development.	
**Institutional Integration and Policy Alignment** Strategic fit, system legitimacy, policy resonance	**Sustainability:** Integration into the stable operations of national health/sport systems.	Align initiative with existing national sport and health policy priorities to support institutional recognition and system legitimacy.	**Sustaining Condition (Schedule It)** Necessary to move from a locally valued practice to a high priority intervention nationally.
	**Penetration:** Reach within the FAI and HSE systems.	Develop standardised external engagement materials enabling partner organisations to initiate local delivery pathways with reduced central coordination.	

This table synthesises prioritised determinants and corresponding implementation strategies across ecological levels to support the national replication of the FC initiative.

The implementation strategies prioritised across all ecological levels focus on a shift toward formalisation: specifically, reducing the reliance on informal coordination while distributing leadership and strengthening both administrative and digital systems. This approach also seeks to secure consistent facility access and establish delivery standards that align the FC initiative more closely with existing sport and health frameworks. To clear the path for immediate replication, the “Do It Now” priorities target the most pressing structural bottlenecks, including governance clarity, volunteer coordination workloads, and the foundational IT and facility infrastructure. Furthermore, creating a robust workforce through structured training and induction for Volunteer Game Coordinators emerged as a prerequisite for expansion, ensuring that delivery quality remains consistent across new sites.

Scheduled prerequisites focused on further strengthening organisational capacity and governance arrangements required to support expansion, while sustaining conditions related to longer-term institutional integration, partnership development, and evaluation systems capable of supporting ongoing learning and funding justification.

Collectively, these strategies represent targeted responses to determinants identified in PRACTIS Step 3 and reflect areas where the subsequent ISAT appraisal indicated organisational and infrastructural considerations relevant to scale-up.

### 3.5 Integrated scalability appraisal (ISAT)

**Figure 1:**
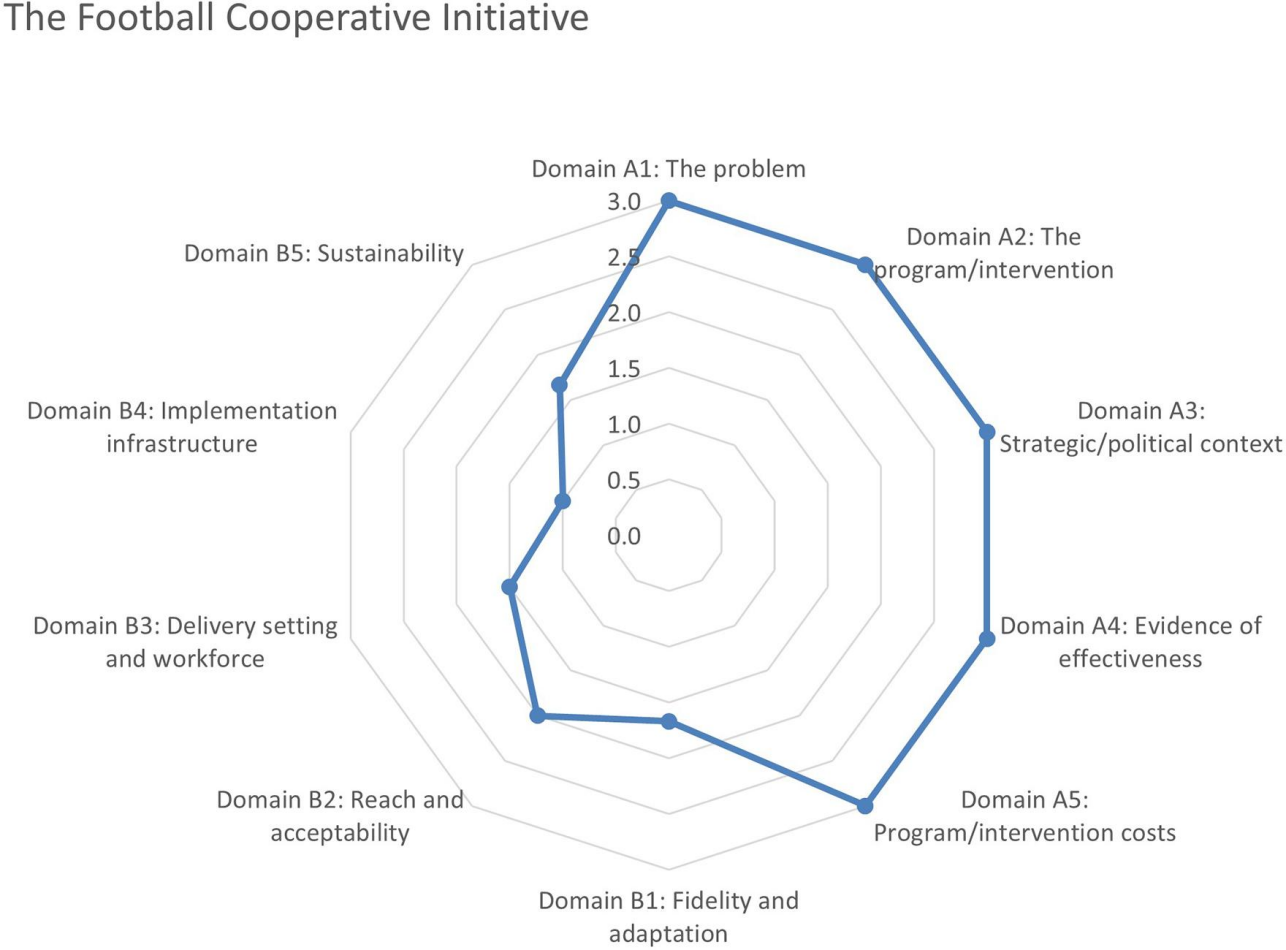
ISAT (69) scalability appraisal across intervention domains for the Football Cooperative initiative.

These ISAT findings (Figure 1) are consistent with earlier results demonstrating alignment between the FC initiative and national men's health and sport participation priorities. They also align with evidence of positive participant health and wellbeing outcomes and sustained engagement across delivery sites (13, 59–61).

Moderate readiness was observed across domains relating to fidelity, reach, and workforce capacity. While guiding principles and delivery values are clearly articulated and participant acceptability is high, replication currently depends on informal knowledge transfer and sustained volunteer coordination. As identified in Sections 3.3 and 3.4, relational facilitation practices, cultural cohesion, and volunteer workload operate as cross-level mechanisms shaping implementation feasibility. This highlights the need for structured supports to maintain delivery quality during expansion.

Lower readiness was identified in domains relating to implementation infrastructure and long-term sustainability. Determinant analysis highlighted a reliance on manual administrative processes, informal digital communication systems, and concentrated operational responsibility within a small number of roles. This indicates limited implementation infrastructure to support coordinated replication across multiple sites. These structural features indicate potential constraints on scalability without strengthened organisational systems, distributed leadership arrangements, and formalised capacity-building mechanisms.

Consistent with ISAT guidance, the appraisal does not generate a single overall scalability score but instead identifies relative strengths and constraints to inform implementation planning. Overall, the profile synthesises preceding findings to highlight contextual strengths alongside organisational and infrastructural constraints relevant to future scale-up of the FC initiative.

## 4 Discussion

Findings from this study highlight how implementation of the FC initiative is shaped by interacting influences across participant, provider, organisational, and system levels, which informed the development of prioritised strategies for scale-up. Consistent with IS evidence highlighting persistent research-to-practice gaps (53, 55), findings suggest that strong intervention outcomes alone are insufficient for scale-up. Alignment between relational engagement processes and organisational capacity is also required. Similar challenges have been observed in gender-responsive initiatives such as SFL and FFIT, where sustained scale-up required governance and workforce strengthening alongside strong participant acceptability (50, 55).

The ISAT appraisal demonstrated strong readiness across problem definition and evidence domains, reflecting the initiative's alignment with established gender-responsive approaches (15, 16). However, lower readiness in workforce and infrastructure domains aligned with determinants relating to volunteer workload and informal coordination. By linking the ecological determinants with ISAT readiness domains and prioritised implementation strategies, this study provides an insight into how community-based men's health initiatives can address structural constraints on scalability.

### 4.1 Prioritised strategies as immediate implementation responses

The prioritised implementation strategies in Table 5 outline the operational conditions needed to stabilise delivery of the FC initiative before expansion to additional sites. Those strategies were derived by integrating the multilevel determinant analysis with the ISAT appraisal and therefore reflect implementation requirements across participant, provider, organisational, and system levels. This approach aligns with recent efforts within IS to identify and prioritise multi-level strategies capable of supporting implementation in complex, community-based settings where delivery systems are often decentralised and resource constrained (78).

The FC initiative was consistently described across stakeholder accounts, as a highly acceptable participation model that engages men through an informal and socially supportive football environment. However, the determinant synthesis indicated that sustaining and replicating this model requires organisational and system structures that extend beyond the organisation of weekly games. Similar patterns have been observed in community sport and mental health promotion initiatives, where sustained delivery depends not only on participant engagement but also on organisational leadership, partnership networks, and adequate human and financial resources (79). The prioritised strategies therefore identify the implementation conditions required to stabilise delivery while preserving the relational participation environment that underpins engagement.

**Figure 2:**
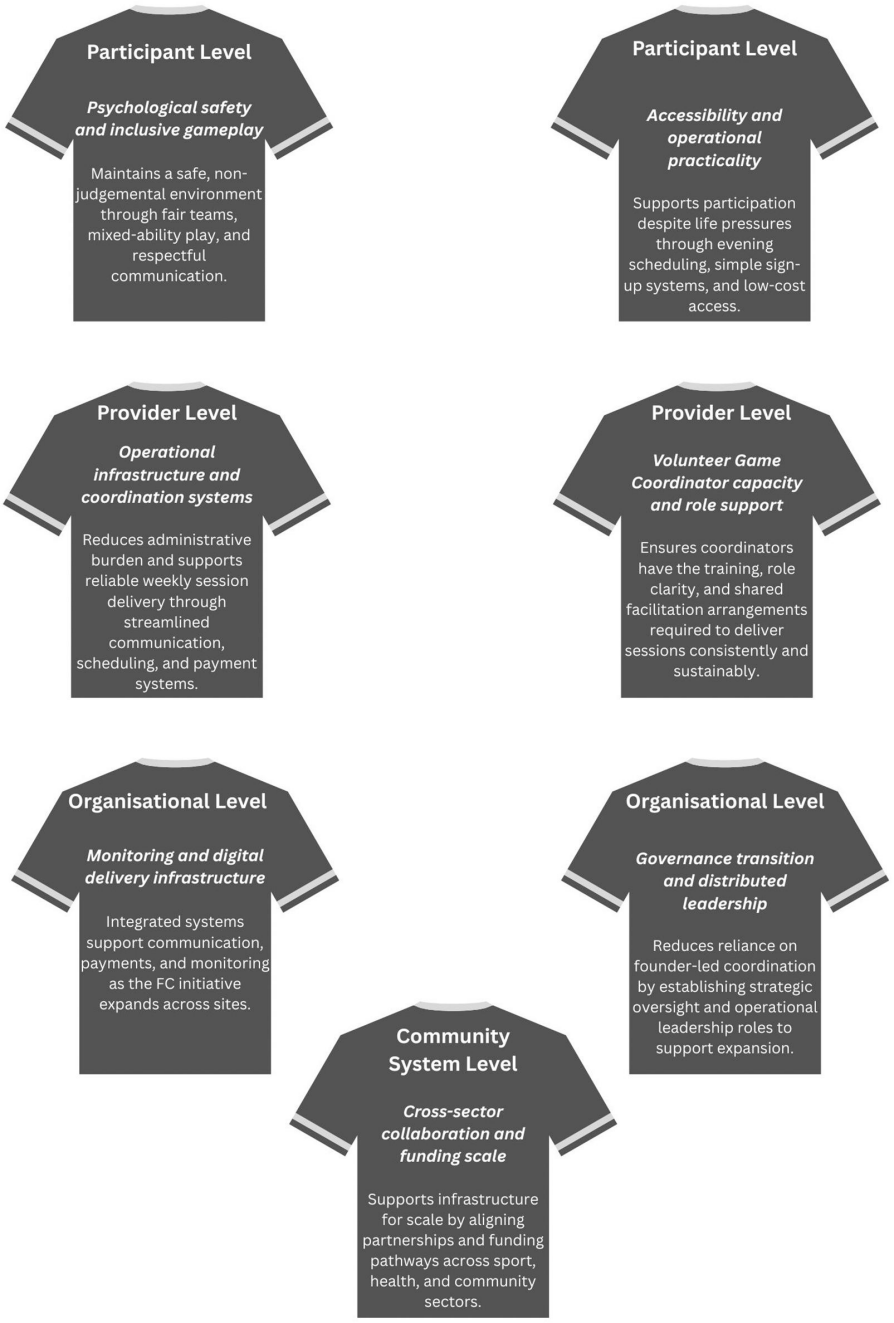
Core implementation conditions for initiating FC replication (“Do It Now” priorities).

Seven strategies were identified as immediate “Do It Now” priorities, representing foundational implementation conditions supporting feasibility, acceptability, and sustainability outcomes across ecological levels. At the participant level, maintaining accessible participation structures and protecting psychologically safe gameplay environments appear central to enabling initial engagement and continued attendance. At the provider level, strengthening digital coordination systems and structured support for VCs is likely necessary to support reliable session delivery and reduce the administrative workload associated with weekly coordination. At the organisational level, governance transition and infrastructure development point to the need for distributed leadership and reduced reliance on a small number of individuals responsible for strategic oversight and operational coordination. Finally, at the community and system level, cross-sector collaboration and diversified funding pathways were identified as mechanisms for embedding the FC initiative within broader sport, health, and community participation infrastructures. These seven “Do It Now” priorities are summarised visually in Figure 2 as the core implementation conditions required before expansion to additional FC initiative sites.

Addressing these foundational implementation conditions represents an important step in moving the FC initiative from a locally delivered initiative toward a scalable participation model. Implementation science research consistently highlights that initiatives demonstrating strong participant acceptability require accompanying organisational and system conditions to sustain delivery and enable scale-up (53–55). Emerging evidence further emphasises the importance of stable funding arrangements, leadership structures, and community “soft infrastructure” such as local partnerships and relational networks in supporting the sustainability of community-based initiatives (80–82). In this context, the prioritised strategies function as stabilisation mechanisms for the implementation system, strengthening infrastructure while maintaining the social and cultural features that make the FC initiative attractive to participants.

### 4.2 Relational fidelity and psychological safety as core implementation mechanisms

Findings from the determinant analysis indicated that sustained participation in the FC initiative was closely linked to the presence of a psychologically safe and socially supportive playing environment. Participants described sessions as welcoming, non-judgemental, and accommodating of mixed ability levels, enabling men with different levels of fitness, football experience, and confidence to participate without fear of embarrassment or exclusion. Similar engagement dynamics have been observed in gender-responsive men's health initiatives delivered through sport settings, where informal participation environments help reduce stigma and create socially acceptable entry points for health engagement (15, 16). These findings suggest that psychological safety operates as a central mechanism underpinning engagement within the FC initiative.

Psychological safety in sport has been conceptualised as a group-level climate in which individuals feel able to participate and express themselves without fear of negative social consequences (83). Within the FC initiative, engagement appeared to be sustained not through structured health messaging but through the social experience of playing football in an environment where enjoyment, fairness, and inclusion were prioritised over competitive performance. Similar dynamics have been observed in football-based health initiatives such as Football Fans in Training, where the cultural familiarity of football and supportive peer environments facilitate sustained engagement among men who may otherwise avoid traditional health services (21, 57).

The findings also highlight the importance of relational fidelity in programme implementation. While conventional interpretations of fidelity often emphasise adherence to predefined intervention components, the FC initiative relies on maintaining the relational conditions that enable mixed-ability participation and inclusive gameplay. Realist analyses of sport-based health initiatives similarly emphasise safe peer environments and skilled facilitation as mechanisms through which programmes influence motivation and participation (21).

Within this context, VCs play a key facilitative role. Beyond coordinating logistics, they help maintain session tone by organising mixed-ability teams, reinforcing inclusive norms, and intervening when games become overly competitive. As the FC initiative expands, preserving these relational conditions will be as important as replicating structural delivery components. Implementation strategies therefore emphasise minimum delivery standards, VC training, and peer learning to support consistent facilitation of inclusive gameplay across sites. These questions also deserve direct reflection in the context of scale-up: can the qualities of informality, local responsiveness, and peer-regulated culture that appear central to the FC initiative's engagement survive formalisation, and if so, how? The argument advanced here, and consistent with the findings in Section 3.3.5, is that formalisation of organisational systems need not compromise informal participation cultures if a clear distinction is maintained between the two. Trust and local relevance are preserved not by resisting governance development but by ensuring that governance structures are designed to protect and resource the conditions of inclusive delivery rather than to standardise or professionalise participation itself. Community ownership is maintained through distributed leadership models in which those closest to delivery retain meaningful agency over how games are organised and facilitated, with formal structures providing support rather than direction. In this sense, the relational qualities central to the FC initiative's success are not casualties of scale but conditions that implementation infrastructure must be explicitly designed to sustain.

### 4.3 Operational infrastructure and coordination burden

Although engagement in the FC initiative was strong, scalability was constrained by coordination demands that were largely invisible to participants but substantial for those responsible for organising delivery. Manual attendance tracking, communication management, and session oversight were largely embedded within volunteer roles, rendering the administrative infrastructure both invisible and fragile. The lower readiness scores in workforce capacity and infrastructure, identified through the ISAT, point directly to this heavy concentration of operational responsibility within a few key roles.

These findings indicate that the hidden coordination burden functions as a structural constraint on scalability rather than a minor operational inefficiency. Reliance on informal, founder-led and volunteer-sustained systems exposes the initiative to implementation fatigue, where cumulative administrative demands threaten continuity as delivery expands. Scalability challenges therefore stem not from deficits in acceptability or effectiveness, but from misalignment between delivery demands and organisational supports. Research on grassroots sport volunteering similarly highlights how administrative responsibilities and coordination demands frequently generate frustration among volunteers, particularly when delivery systems rely on informal or fragmented organisational structures (84).

Comparable challenges have been observed in other community-based initiatives. In Sheds for Life, for example, institutional capacity constrained sustainability during programme expansion (49, 50). Within sport organisations more broadly, sustaining volunteer engagement has been shown to depend not only on individual motivation but also on organisational systems that support coordination, role clarity, and manageable workloads (85). In this context, the transition toward digital coordination systems and distributed leadership represents not merely an operational upgrade but a theory-informed requirement to stabilise infrastructure and prevent abandonment. Strengthening workforce and system supports is therefore a prerequisite for maintaining Feasibility and Sustainability as the initiative scales.

### 4.4 Contribution beyond the FC initiative: institutional integration and implementation blueprint

The findings also offer broader insight into how community-based men's health interventions move from locally effective models to institutionally sustained systems of delivery. While culturally embedded participation “hooks” such as football enhance engagement and acceptability, scalability depends on organisational and system capacity to maintain these conditions over time. Engagement alone does not secure sustainability, a limitation repeatedly identified in community-based men's health promotion where popular, activity-based formats often struggle to endure beyond short-term project funding cycles (23, 86).

The research demonstrates that gender-responsive engagement mechanisms must be supported by infrastructure capable of preserving relational fidelity at scale. Without distributed leadership, digital coordination systems, and formalised governance roles, locally effective models remain structurally fragile as delivery expands across sites. This reinforces implementation science evidence that programme effectiveness does not automatically translate into scalability without organisational development and system capacity (67, 87). In doing so, the findings extend existing work by illustrating the relational and governance conditions required to maintain engagement mechanisms as scale increases (49, 50, 88).

The governance transition identified in this study reflects more than organisational maturation. Recreational sport initiatives frequently operate outside formal governance and funding structures, limiting legitimacy and long-term resourcing (23). Institutional integration therefore functions as a mechanism for system recognition, enabling alignment with policy priorities and sustained investment. Comparable scale-up pathways are evident in programmes such as FFIT and SfL, where long-term sustainability depended on embedding delivery within established organisational structures and securing central funding support (49, 50, 57). It is worth noting, however, that FFIT's scale-up was underpinned by mechanisms specific to its professional club context, including institutional trust in professional football organisations, structured behaviour change programming, trained coach delivery, and the mobilisation of gendered club identity as a driver of engagement and sustained participation (44–46, 57). These mechanisms are not directly transferable to the FC initiative's community-based, recreationally anchored model, and the FC initiative's scale-up therefore requires a distinct set of implementation conditions, as identified in this study, rather than a replication of the FFIT governance pathway. The present study therefore illustrates how community-based men's health initiatives can move from *ad hoc*, locally sustained provision toward routinised, system-governed delivery while retaining men-friendly practice.

This paper also offers a transferable implementation blueprint for community-based sport and health initiatives. While culturally familiar participation formats may successfully attract participants, sustained scale-up depends on organisational infrastructure capable of supporting delivery across multiple sites. By linking ecological determinant analysis with structured strategy development through PRACTIS and readiness appraisal through ISAT (67, 69, 89), this research demonstrates how community-based interventions can move from relationally effective local practice toward institutionally supported and scalable systems of delivery, addressing wider calls for prospective, tool-guided scalability planning in physical activity and men's health promotion (90, 91). This need for rigorous external evaluation support is consistent with challenges identified across football club community trust programmes more broadly, where sustaining funding and demonstrating impact through robust evaluation have been identified as persistent implementation barriers (92).

### 4.5 Strengths and limitations

This study drew on multilevel perspectives across participant, provider, organisational, and community or system stakeholders both within and outside of the FC initiative, enabling examination of implementation determinants across ecological levels and across comparable sport-based health initiatives. Integration of CFIR-informed analysis, PRACTIS planning, and ISAT appraisal supported a transparent progression from determinant identification to prioritised strategy development. The combination of stakeholder perspectives, observational insights, and prior evaluation evidence strengthened analytic triangulation and allowed implementation influences to be examined across delivery, organisational, and wider system contexts. Bringing these elements together within a single study also enabled qualitative implementation insights to be translated into a structured implementation strategy to support scale-up planning.

The findings are grounded in the context of a community-based football initiative, and implementation conditions may vary across different organisational or sport settings. However, the study also engaged stakeholders and evidence from comparable initiatives operating at larger scale, supporting interpretation of the findings beyond a single initiative context. In addition, the ISAT appraisal involved structured expert judgement informed by qualitative and evaluation evidence, reflecting the interpretive nature of scalability assessment.

A further limitation relates to the organisational-level analysis, which primarily drew on longitudinal interviews with a single organisational lead responsible for development and coordination of the FC initiative. While this provided detailed insight into organisational decision-making and strategic development over time, it also reflects the current leadership structure of the initiative. This limitation was partially mitigated through triangulation with provider, community or system stakeholders, and advisory board members who contributed perspectives on governance, partnership development, and system integration. These complementary accounts enabled examination of organisational influences beyond a single viewpoint while retaining the depth of insight provided by the organisational lead.

## 5 Conclusion

Scaling community-based men's health initiatives requires alignment between relational engagement mechanisms, organisational infrastructure, and wider system support. While culturally embedded “hooks” can generate strong acceptability and reach, sustainability requires parallel development of governance, workforce capacity, and implementation systems. The FC initiative demonstrates that psychological safety and peer-led engagement can attract sustained participation, but the ISAT appraisal highlights that infrastructure and distributed leadership are necessary to support delivery at scale. By linking ecological determinant analysis, structured strategy development, and scalability appraisal, this study identifies the organisational, workforce, and system conditions required to stabilise the FC initiative as it expands across new sites. These findings highlight that sustaining the relational participation environment central to the FC initiative requires parallel investment in governance structures, coordination systems, and cross-sector partnerships. They may also inform the scale-up of other community-based men's health initiatives beyond locally effective delivery.

## Data Availability

The raw data supporting the conclusions of this article will be made available by the authors, without undue reservation.
